# Comparison of Sutureless and Conventional Laparoscopic Partial Nephrectomy: A Propensity Score-Matching Analysis

**DOI:** 10.3389/fonc.2021.649356

**Published:** 2021-03-08

**Authors:** Feng Zhang, Shuang Gao, Yiqiao Zhao, Bin Wu, Xiaonan Chen

**Affiliations:** ^1^Department of Urology, Shengjing Hospital of China Medical University, Shenyang, China; ^2^Department of Pathology, The People's Hospital of Liaoning Province, Shenyang, China

**Keywords:** hemostasis, laparoscocpy, nephrectomy, organ sparing treatments, sutureless

## Abstract

**Objective:** To compare the functional outcome, safety and efficacy of sutureless and conventional laparoscopic partial nephrectomy.

**Methods:** After the inclusion and exclusion criteria were applied, our study reviewed 379 patients with T1 stage renal tumors. We applied propensity score matching (PSM) to limit potential baseline confusion. Perioperative and functional outcomes between sutureless laparoscopic partial nephrectomy (sLPN) and conventional laparoscopic partial nephrectomy (cLPN) groups were compared and analyzed before and after PSM.

**Results:** Of our 379 patients with T1 stage renal tumors, 199 and 180 were identified in the cLPN and sLPN groups, respectively. After applying PSM with preoperative features, 116 patients in the cLNP group were paired to 116 patients in the sLNP group. We found that all differences in preoperative baseline characteristics disappeared. All the preoperative characteristics (age, gender, tumor diameter, RENAL nephrometry score, side, preoperative eGFR, hypertension, diabetes mellitus, ASA score) were not statistically different between the two groups. The operative time (OT) (*p* < 0.001) and warm ischemia time (WIT) (*p* < 0.001) of the sLPN group were of shorter duration than that of the cLPN group. The eGFR baseline was almost equal, but there was a statistically smaller decrease in eGFR in the sLPN than in the cLPN group 1 week after surgery (14.3 vs. 7.4, *p* < 0.001) and after 6 months (11.9 vs. 5.0, *p* < 0.001). After both preoperative features and WIT were included in PSM, fifty-one pairs of patients were identified between the groups, the WIT difference between them disappeared, while the decrease in eGFR between the groups remained as it was previously at 1 week (15.4 vs. 8.6, *p* < 0.001) and at 6 months (13.0 vs. 6.2, *p* < 0.001).

**Conclusion:** Sutureless laparoscopic partial nephrectomy is as safe and effective as conventional laparoscopic partial nephrectomy, and compared to cLPN, sLPN can effectively reduce the WIT, retain more renal parenchyma and protect renal function.

## Introduction

With the increasing popularity of ultrasound or CT examination, the incidence rate of diagnosis of renal tumors is increasing year by year. Partial nephrectomy is recommended for T1 stage renal tumors because it has a better oncological and functional prognosis compared with radical nephrectomy (1). The ultimate goal of the PN is, negative surgical margins, functional preservation and complication-free recovery (2). The preservation of renal function has three main benefits; the time of warm ischemia, the preservation of the normal kidney, and the reconstruction of the renal remnant (3). In conventional partial nephrectomy, the first step in the procedure is to block the renal pedicle so as to provide a completely bloodless surgical field of vision for the operation (4). The occlusion of the renal pedicle, however, inevitably results in so-called warm ischemia time (WIT), in which injury to the kidney tissue takes place (5). In addition to the WIT, the size of tumor resection and the preservation of renal parenchyma also play important roles in the preservation of renal function. At present, it is believed that the enucleation of the renal tumor can achieve an optimal oncological result provided the capsule of the tumor is intact (1). Bahler and Sundaram (3) stated that the reconstruction of renal parenchyma is more important than the effect of WIT on renal function.

We previously reported a method of sutureless and clampless laparoscopic partial nephrectomy with monopolar coagulation, and in the early cases where the procedure was undertaken, this method proved to be both feasible and safe (6). In this study, we aimed to compare the results of sutureless laparoscopic partial nephrectomy with those of conventional laparoscopic partial nephrectomy, using propensity score matching (PSM), and including perioperative data and functional results.

## Patients and Methods

### Data Acquisition

Our study data were obtained from a retrospective maintenance database, following approval by the institutional review board and the ethics committee of Shengjing Hospital (No.2018PS012J), all patients provided written scientific ethics consents. From February 2015 to February 2018, all patients who underwent laparoscopic partial nephrectomy (LPN), whether sutureless or conventional, for T1 renal tumor, were identified. Those patients with multiple renal tumors or with preoperative renal dysfunction were excluded. All patients had been diagnosed with T1 renal tumor for the first time. A total of 379 patients were classified into either the conventional laparoscopic partial nephrectomy (cLPN) (*n* = 199) group or the sutureless laparoscopic partial nephrectomy (sLPN) (*n* = 180) group. The patients' demographic and clinical information was recorded. The RENAL nephrometry scores were assessed by a single doctor, based on perioperative CT scans. The LPNs were all performed by experienced surgeons, from a group of four surgeons in all. The surgical approach, whether transperitoneal or retrotransperitoneal, was chosen depending on the location of the tumor and the preference of the individual surgeon.

Clinical characteristics included: age, gender, hypertension, diabetes mellitus, tumor size, RENAL score, and preoperative estimated glomerular filtration rates (eGFR), American Society of Anesthesiologists (ASA) score. Perioperative outcomes included: surgical approach, operating time (OT), warm ischemia time (WIT), estimated blood loss (EBL), positive surgical margin, postoperative hospital stay, and postoperative complications. Renal functional results were assessed by eGFR within postoperative 1 week and again at 6 months.

### Propensity Score-Matching

**Figure 1:**
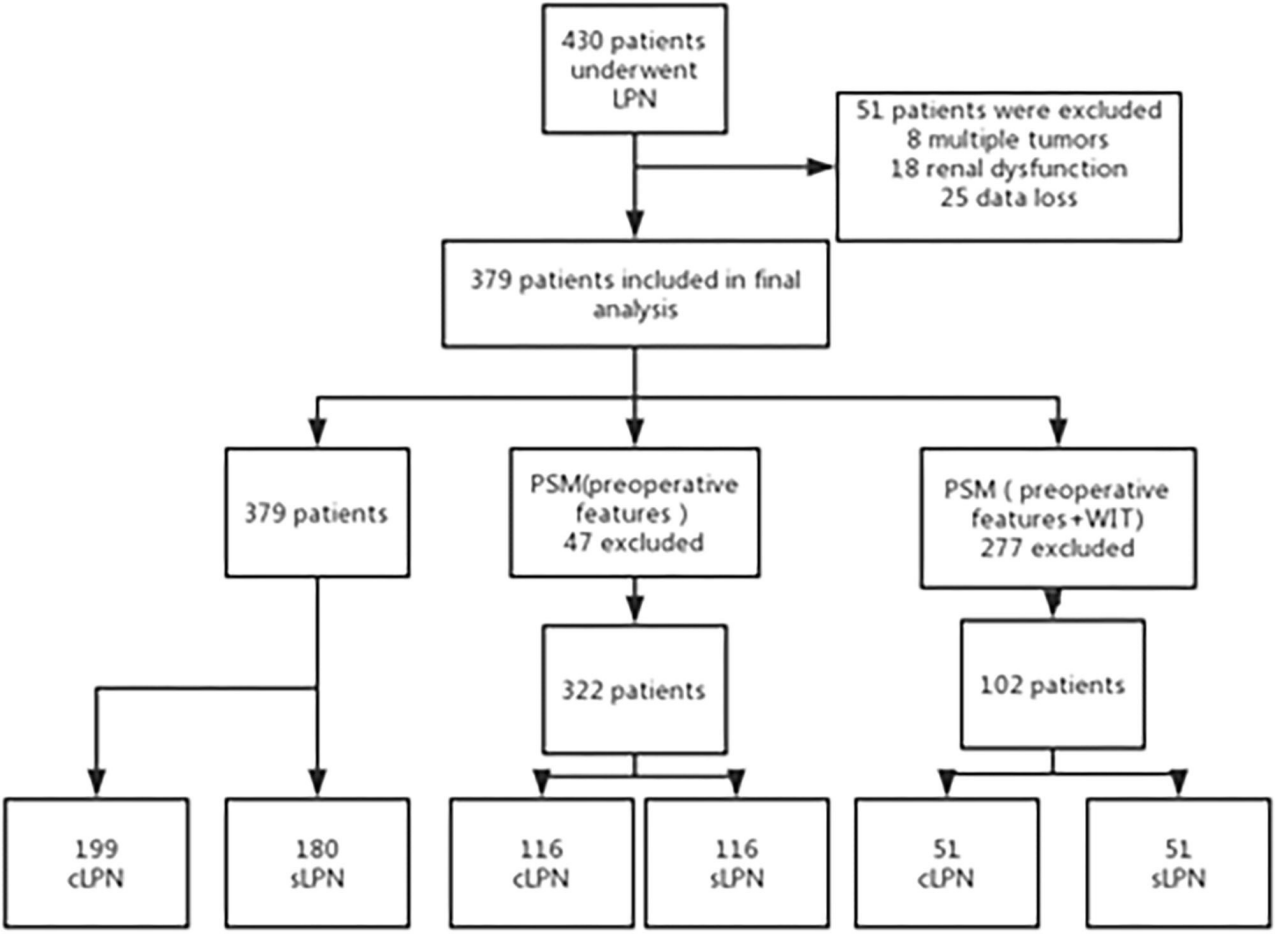
Flow chart of the study.

To improve the accuracy of our conclusions, we used the propensity score-matching (PSM) method to eliminate baseline differences between the sLPN and cLPN groups. Multivariate logistic regression analysis was performed to determine propensity scores based on all perioperative features. Preoperative features are important basis for choosing surgical procedure, so we excluded the intra- and postoperative outcomes from the PSM process. In accordance with the nearest neighbor matching method, one hundred and sixteen patients in the sLPN group were paired to 116 patients in the cLPN group in a 1:1 ratio. In order to eliminate the effect of WIT on renal function, we took WIT into account in the PSM. Fifty-one patients in the sLPN group were paired to 51 patients in the cLPN group in a 1:1 ratio (Figure 1).

### Brief Description of Surgical Techniques

Sutureless LPN has been previously described (6). A three-trocar technique was used via the transperitoneal or retroperitoneal approach. Gerota's (anterior renal) fascia and perirenal fat was transversed so as to locate the tumor. The fat tissue overlying the tumor was conserved where it is feasible. The renal hilar was occluded when necessary. The tumors were usually removed with a cold scissor and bluntly enucleated with a clamp or suction aspirator outside the tumor's pseudocapsule. The monopolar hook was used for coagulation when hemorrhage appeared. Repeated monopolar coagulation was performed on the tumor bed after tumor excision. In 145 of all 180 cases, n-butyl-cyanoacrylate (NBCA) was sprayed for hemostasis.

Conventional laparoscopic partial nephrectomy was carried out according to the process previously described (4). Tumor resection was outside the pseudocapsule in the same way as the sutureless partial nephrectomy, the difference was that after tumor removal, split blood vessels and collecting system were fixed with running sutures to secure hemostasis and water-tight closure with a 3-0 barbed suture. The renal parenchyma was running sutured in the second layer with a 2-0 barbed suture.

### Statistical Analysis

Statistical analyses were performed using SPSS 22.0 for Windows (SPSS, Inc., Chicago, IL, USA). Continuous variables with a normal distribution were recorded as the mean ± SD, and Student's *t*-test was used to compare the outcomes. Non-normal continuous variables were recorded as the median (interquartile range). Mann-Whitney U test was used to compare the outcomes. Categorical variables were recorded as numbers (percentage), Pearson's chi-square test was used to compare the outcomes. Values of *p* < 0.05 (two-tailed) were considered to be statistically significant.

## Results

### Preoperative Characteristics

A total of 379 patients, including 180 sLPN and 199 cLPN patients, were enrolled in the study. Patient demographics and preoperative features are listed in Table 1. Before PSM, the mean age of the cLPN group was higher than that of the sLPN group (*p* = 0.005). Other variables (gender, diameter of tumor, RENAL nephrometry score, E score, N score, side, preoperative eGFR, hypertension, diabetes mellitus, ASA score) had no statistically differences between the two groups. After PSM, the statistically significant age differences between the two groups was no longer evident (Table 1).

**Table 1:** Preoperative features of the patients before and after PSM.

	**Before PSM**			**After PSM**			**After PSM (including WIT)**		
**Characteristics**	**cLPN**	**sLPN**	***p*****-value**	**cLPN**	**sLPN**	**p-value**	**cLPN**	**sLPN**	***p*****-value**
Patients (*n*)	199	180		116	116		51	51	
Age (years)	58 (27–82)	54 (21–86)	0.005	57 (27–78)	57.5 (21–86)	0.538	53.1 (27–77)	55.6 (31–75)	0.357
Gender			0.952			0.787			0.316
Male (%)	120 (63.5)	108 (60)		73 (62.9)	71 (61.2)		27 (52.9)	32 (62.7)	
Female (%)	79 (36.5)	72 (40)		43 (37.1)	45 (38.8)		24 (47.1)	19 (37.3)	
Diameters of tumor (cm)	3.1 (1.0–7.0)	3.0 (1.0–7.0)	0.06	3.0 (1.5–7.0)	3.0 (1.0–7.0)	0.934	3.4 (1.5–6.0)	3.6 (1.4–6.7)	0.381
RENAL score (median)	6	6	0.104	6	6	0.258	6	7	0.356
Low (4–6) (%)	118 (59.3)	98 (54.4)		69 (59.5)	80 (69)		24 (47.1)	28 (54.9)	
Medium (7–9) (%)	75 (37.7)	63 (35)		43 (37.1)	31 (26.7)		23 (45.1)	14 (27.5)	
High (10–12) (%)	6 (3)	19 (10.6)		4 (3.4)	5 (4.3)		4 (7.8)	9 (17.6)	
E score			0.0001			0.33			0.078
1 (%)	99 (49.7)	69 (38.3)		51 (44)	60 (51.7)		14 (27.5)	22 (43.1)	
2 (%)	95 (47.7)	85 (47.2)		61 (52.6)	50 (43.1)		35 (68.6)	24 (47.1)	
3 (%)	5 (2.5)	26 (14.4)		4 (3.4)	6 (5.2)		2 (3.9)	5 (9.8)	
*N* score			0.109			0.893			0.882
1 (>7 mm) (%)	125 (62.8)	111 (61.7)		78 (67.2)	81 (69.8)		30 (58.8)	31 (60.8)	
2 (4–7 mm) (%)	57 (28.6)	42 (23.3)		28 (24.1)	25 (21.6)		12 (23.5)	10 (19.6)	
3 (<4 mm) (%)	17 (8.5)	27 (15)		10 (8.6)	10 (8.6)		9 (17.6)	10 (19.6)	
Side			0.142			0.792			0.313
Left (%)	109 (54.8)	85 (47.2)		63 (54.3)	61 (52.6)		33 (64.7)	28 (54.9)	
Right (%)	90 (45.2)	95 (52.8)		53 (45.7)	55 (47.4)		18 (35.3)	23 (45.1)	
Preoperative eGFR (ml/min)	91.2 ± 11.9	94.5 ± 17.1	0.906	94.1 ± 11.1	94.7 ± 17.4	0.325	90.5 ± 10.4	90.3 ± 16.4	0.924
Hypertension (%)	28 (14.1)	27 (15)	0.797	15 (12.9)	20 (17.2)	0.359	5 (9.8)	9 (17.6)	0.25
Diabetes mellitus (%)	19 (9.5)	29 (16.1)	0.617	13 (11.2)	13 (11.2)	1	4 (7.8)	6 (11.8)	0.505
ASA score (≥3) (%)	8 (4%)	7 (3.9)	0.948	3 (2.6)	5 (4.3)	0.472	2 (3.9)	2 (3.9)	1

*PSM, Propensity Score Matching; WIT, Warm Ischemia Time; cLPN, convential Laproscopic Partal Nephrectomy; sLPN, sutureless Laproscopic Partal Nephrectomy; ASA, American Society of Anesthesiologists*.

### Operative and Perioperative Characteristics

Operative and perioperative characteristics are listed in Table 2. After PSM, the operative time was significantly shorter in the sLNP group than in the cLPN group (135.8 vs. 168.2 min, *p* < 0.001). Median WIT was also significantly shorter in the sLPN group than in the cLPN group (6.6 vs. 22 min, *p* < 0.001). Other variables (EWL, tumor location, transfusion, conversion to open surgery, postoperative hospital stay, PSM, local recurrence, pathology) were not statistically different between the two groups. But after PSM within WIT, the differences in OT and WIT were not evident. The pathological results are listed in Table 3. There was no statistical difference in all the results between the two groups.

**Table 2:** Intra-and postoperative features after PSM.

	**After PSM**			**After PSM within WIT**		
**Characteristics**	**cLPN (116)**	**sLPN (116)**	***p*****-value**	**cLPN (51)**	**sLPN (51)**	***p*****-value**
Operative time (min)	168.2 (60–300)	135.8 (40–250)	0.0001	153.1 (60–260)	158.6 (50–300)	0.614
WIT (min)	22 (0–45)	6.6 (0–40)	0.0001	21.5 (0–30)	21.2 (0–41)	0.888
Mean estimated blood loss (ml)	161 (20–1000)	154 (10–800)	0.501	200 (40–1000)	208 (10–1000)	0.835
Parahilar tumor (%)	14 (12.1)	15 (12.9)	0.85	7 (13.7)	9 (17.6)	0.58
Endophytic tumor (%)	4 (3.4)	6 (5.2)	0.517	2 (3.9)	5 (9.8)	0.24
Transfusion (%)	8 (6.9)	9 (7.8)	0.801	3 (5.9)	2 (3.9)	0.647
Conversion to open (%)	2 (1.7)	1 (0.9)	0.501	1 (2.0)	1 (2.0)	1
Postoperative hospital stay (day)	10.1 (5–25)	10.3 (4–36)	0.178	19.8 (12–32)	19.5 (11–40)	0.77
Positive surgical margin (%)	3 (2.6)	2 (1.7)	0.651	2 (3.9)	0 (0)	0.153
Local recurrence (%)	2 (1.7)	1 (0.9)	0.501	0 (0)	0 (0)	1

*PSM, Propensity Score Matching; WIT, Warm Ischemia Time; cLPN, convential Laproscopic Partal Nephrectomy; sLPN, sutureless Laproscopic Partal Nephrectomy*.

**Table 3:** Pathological results of the patients.

	**Before PSM (%)**			**After PSM (%)**			**After PSM (including WIT) (%)**		
	**cLPN**	**sLPN**	***p*****-value**	**cLPN**	**sLPN**	***p*****-value**	**cLPN**	**sLPN**	**p-value**
Histology			0.211			0.508			0.635
AML	28 (14.1)	34 (18.9)		18 (15.5)	23 (19.8)		12 (23.5)	8 (15.7)	
Oncocytoma	10 (5.0)	4 (2.2)		2 (1.7)	2 (1.7)		0 (0)	0 (0)	
Clear cell	142 (71.4)	126 (70)		85 (73.3)	77 (66.4)		33 (64.7)	38 (74.5)	
Papillary	7 (3.5)	9 (5)		4 (3.4)	8 (6.9)		3 (5.9)	2 (3.9)	
Chromophobe	8 (4)	2 (1.1)		5 (4.3)	2 (1.7)		1 (2.0)	0 (0)	
Others	4 (2)	5 (28.8)		2 (1.7)	4 (3.4)		2 (3.9)	3 (5.9)	

*PSM, Propensity Score Matching; WIT, Warm Ischemia Time; cLPN, convential Laproscopic Partal Nephrectomy; sLPN, sutureless Laproscopic Partal Nephrectomy; AML, angiomyolipoma*.

### Perioperative Renal Function

The pre- and post-operative renal function results are listed in Table 4. The median eGFR was 94.1 and 94.7 ml/min in the matched cLPN group and the matched sLPN group after PSM (without WIT), respectively. The eGFR was 90.5 and 90.3 ml/min in the matched cLPN group and the matched sLPN group after PSM (within WIT), respectively. However, following PSM, the changes in eGFR were found to be statistically smaller in the sLPN group than in the cLPN group 1 week or at 6 months after surgery.

**Table 4:** Preoperative eGFR and eGFR changes after PSM.

	**After PSM**			**After PSM (including WIT)**		
	**cLPN**	**sLPN**	***p*****-value**	**cLPN**	**sLPN**	***p*****-value**
Preoperative eGFR (ml/min)	94.1 ± 11.1	94.7 ± 17.4	0.325	90.5 ± 10.4	90.3 ± 16.4	0.924
Changes in eGFR 1 week (ml/min)	14.3 ± 7.6	7.4 ± 4.1	0.0001	15.4 ± 8.1	8.6 ± 5.8	0.0001
Changes in eGFR 6 months (ml/min)	11.9 ± 6.8	5.0 ± 3.6	0.0001	13.0 ± 8.1	6.2 ± 5.8	0.0001

*PSM, Propensity Score Matching; WIT, Warm Ischemia Time; cLPN, convential Laproscopic Partal Nephrectomy; sLPN, sutureless Laproscopic Partal Nephrectomy; eGFR, estimated glomerular filtration rate*.

After PSM, the changes in eGFR in 1 week were significantly different between the cLPN and sLPN groups without WIT (14.3 vs. 7.4, *p* < 0.001), and also at 6 months (11.9 vs. 5.0, *p* < 0.001). After PSM a similar conclusion was obtained within WIT. The changes of eGFR in 1 week were also significantly different between the cLPN and the sLPN group (15.4 vs. 8.6, *p* < 0.001), and also at 6 months (13.0 vs. 6.2, *p* < 0.001).

## Discussion

With better preservation of renal function compared with radical nephrectomy, partial nephrectomy not only reduces cardiovascular disease incidence (7), but also reduces the overall mortality (8). Minimally invasive surgery, in the form of laparoscopic partial nephrectomy, has more advantages in the recovery phase than does open surgery, and the surgical complications are more limited (9).

The ultimate goal of partial nephrectomy is complete resection of tumor, preservation of renal function and a low incidence of postoperative complications (2), With partial nephrectomy, whether in patients with renal insufficiency (10), or in those with normal kidney function (11), the incidence rate of end-stage renal disease is reduced compared to radical nephrectomy. After partial nephrectomy, not only is there better preservation of renal function than with radical nephrectomy, but also the recurrence rate and overall survival rate are better (12). Over 10 years of follow-up data suggest that patients undergoing partial nephrectomy have a better survival rate, better tumor specific survival, and fewer chronic kidney diseases, than those undergoing radical nephrectomy (13). Thus, the latest guidelines recommend that kidney-sparing surgery is better than radical nephrectomy for the management of T1 tumors, both T1a and T1b. With the continuous technology improvements and the more comprehensive understanding of the biological characteristics of renal tumors, an increasing number of doctors prefer partial nephrectomy not only at T1 stage, but also at T2 stage (14), and the outcomes are safe and acceptable (15). Laparoscopic partial nephrectomy (LPN) is the most widely-used method of minimally invasive surgery (16). We included all cases of laparoscopic partial nephrectomy performed in our hospital from February 2015 to February 2018, including both conventional suture cases and sutureless cases. In order to make the renal function of the two groups more comparable, we finally included all T1 cases, excluding multiple tumor cases and cases of preoperative renal insufficiency.

In partial nephrectomy, there are many factors affecting renal function, including the WIT, the preoperative renal function, the extent of preservation of renal parenchyma during the operation, and reconstruction of renal parenchyma (5). For a long time it was believed that the WIT was the most important factor in the preservation of renal function, so that more than 60% of past literature has focused on renal warm ischemia and how to reduce the WIT (3). Thus, 30 min was considered to be the threshold WIT for renal pedicle block (17), and Rod et al. suggest that there was no difference between a WIT < 25 min and zero ischemia (18). However, Thompson et al. considered that reducing the WIT is significant for the preservation of renal function, they published a study entitled “Every minute counts,” in which they found that a decrease of every minute of WIT helped renal function preservation (19). The evidence increasingly shows that renal function benefits from a decrease in WIT. For example, Verze et al. reviewed highly complex renal tumors in both renal pedicle blocked and non-blocked groups. The creatinine in the blocked group increased significantly (20), but after 6 months, there was no significant difference between the two groups. Therefore, much work has been done to reduce the renal WIT, such as the use of zero ischemia laparoscopic partial nephrectomy with preoperative superselective renal artery embolization (21), the precise segmental renal artery clamping technique (22), renal hypothermia with ice slush (23), zero ischemic anatomical partial nephrectomy (24), and so on. In our retrospective analysis of 116 cases of sutureless and conventional laparoscopic partial nephrectomy, we found there was a significant difference in the WIT between the two groups, attributable to the fact that, for sLPN, the sutureless technology significantly reduced the time required for the procedure. In addition, in the sutureless group, tumor resection could be performed by coagulating the tumor bed at the same time as the tumor resection, so the occlusion of the renal pedicle was unnecessary for most of the exophytic tumors. There were significant differences in renal function between the two groups, although whether the benefit in renal function came from the decrease of WIT is unknown. We used another PSM (within WIT) method to re-match the cases to eliminate the effect of WIT between the two groups.

Bahler et al. considered that the reconstruction of renal parenchyma is the most important factor for the preservation of renal function after partial nephrectomy (3), while Zabell et al. concluded that it was the volume and mass of renal parenchyma reserve that were the important factors of renal function, the WIT being merely a secondary factor (25). In conventional partial nephrectomy, bilateral sutures are usually needed. Suturing takes place firstly at the basal layer, in which suturing of the blood vessels and the collection system is usually done. At the second layer, the suturing of renal parenchyma is then done (4). Reducing the number of sutures where possible is considered an important way of preserving renal function (26, 27). According to Huang et al., for T1a stage renal tumors, zero ischemia laparoscopic radio frequency ablation assisted tumor enucleation has obvious advantages over laparoscopic partial nephrectomy in terms of the preservation of renal function (28).

Zhao et al. concluded that there is no clear WIT threshold that has a clear impact on renal function, and the important factor for renal function is the quantity and quality of preserved renal parenchyma (16). Bagheri et al. also deemed the effect of renal parenchyma resection on renal function to be much greater than that of ischemia-reperfusion injury. In our study, after eliminating the difference in WIT between the two groups, we nevertheless concluded that the renal function of the sutureless group was better than that of the sutured group; thus we concluded that the sutureless procedure benefits reserve renal function. This is consistent with the conclusion of the previous systematic review published by Bertolo et al. (27), which found that single-layer suturing produces better outcomes than bilateral suturing in terms of renal function, and that a reduction in suturing confers better protection of renal function. Recently, Jin et al. reported sutureless technique in LPN is safe and feasible, compared with the suture method with shorter WIT, lower AKI rate, but they found no difference of between eGFR decline, which was different in our study (29), and the difference remained even after the factor WIT was removed.

Our study has a number of shortcomings, as follows. We did not report the oncological outcomes of the patients, because we believed the differences between the two groups did not influence the oncological outcomes, since the manner of tumor resection was the same, and the reconstruction of renal parenchyma did not affect the oncological results. Our research was retrospective, not prospective, and inevitably had some selective deviation. The renal function was evaluated by eGFR, which is not exact. Also, we did not assess ipsilateral renal function by means of radionuclide scanning and follow-up time is short. We will try to improve our study design in future study.

In summary, sutureless laparoscopic partial nephrectomy is as safe and effective a treatment as conventional laparoscopic partial nephrectomy; and, compared to cLPN, sLPN can effectively reduce the WIT, retain more renal parenchyma, and protect renal function.

## Data Availability Statement

The raw data supporting the conclusions of this article will be made available by the authors, without undue reservation.

## Ethics Statement

The studies involving human participants were reviewed and approved by the ethics committee of Shengjing Hospital. The patients/participants provided their written informed consent to participate in this study.

## Author Contributions

XC, FZ, and BW conceived and designed the study. SG, YZ, and BW contributed to the analyses of the data. FZ and XC drafted and revised the manuscript. SG and YZ prepared figures and/or tables. All authors read and approved the final manuscript.

## Conflict of Interest

The authors declare that the research was conducted in the absence of any commercial or financial relationships that could be construed as a potential conflict of interest.
